# Supplementary figures and images for: SorCS1-mediated sorting in dendrites maintains neurexin axonal surface polarization required for synaptic function
Source: PLoS Biol. 2019 Oct 28;17(10):e3000466. doi: 10.1371/journal.pbio.3000466 (PMC6837583; doi:10.1371/journal.pbio.3000466)

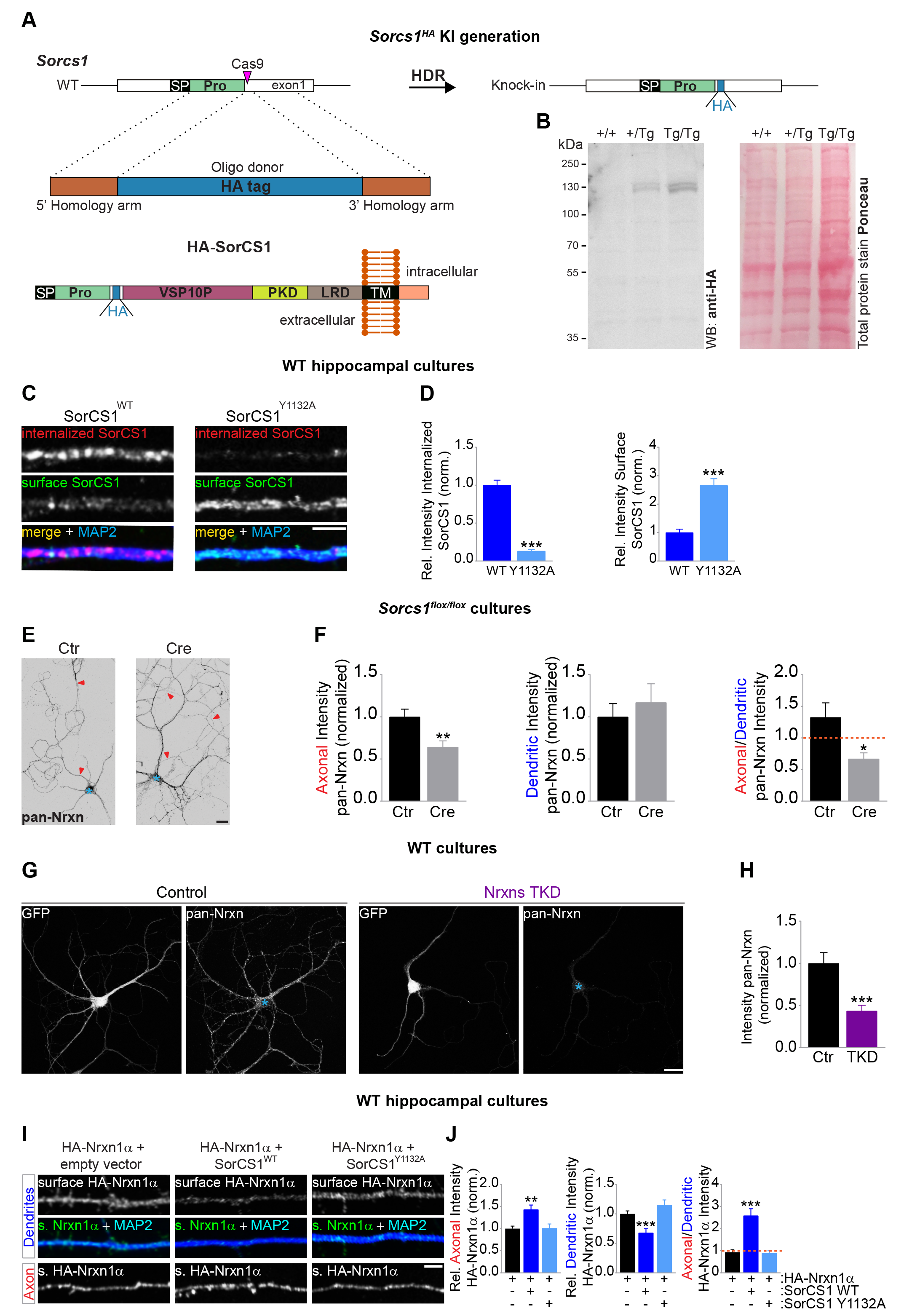

Supplement: S1 Fig — (A) CRISPR/Cas9-mediated generation of Sorcs1HA KI mice. Orange boxes represent the left and right homology arms. Blue box represents the ssDNA donor oligonucleotide containing the HA tag. Schematic representation of SorCS1 protein domain organization is shown to illustrate the HA-tagging of HA-SorCS1 downstream of the second furin cleavage site, right before the VPS10P domain (at the amino acid position 144). (B) Detection of HA-SorCS1 by western blot in total brain extracts prepared from Sorcs1HA KI mice (P60). Total protein staining (Ponceau) shows equal loading between lanes. See S9 Fig for raw uncropped blots. (C) High-zoom images of dendritic internalized (int.) and surface (s.) SorCS1 from DIV9 WT mouse hippocampal neurons expressing HA-tagged WT SorCS1cβ (WT) or Y1132A for 48 h. Live neurons were incubated with an anti-HA antibody and pulse-chased for 20 min. Neurons were immunostained for surface HA-SorCS1 (grayscale and green), internalized SorCS1 (grayscale and red) and MAP2 (blue). (D) Quantification of panel C: internalized SorCS1 fluorescence intensity relative to total levels and normalized to cells expressing WT-SorCS1, surface SorCS1 fluorescence intensity relative to total levels and normalized to cells expressing WT-SorCS1. WT (n = 28 neurons); Y1132A (n = 30). ***P < 0.001 (Mann-Whitney test, 3 independent experiments). (E) DIV10 Sorcs1flox/flox cortical neurons electroporated with EGFP (Ctr) or Cre-EGFP immunostained for pan-Nrxn (grayscale). (F) Quantification of panel E: pan-Nrxn fluorescence intensity in axon and dendrites normalized to cells expressing EGFP and ratio of axonal–dendritic pan-Nrxn intensity. Ctr (n = 30 neurons); Cre (n = 29). *P < 0.05; **P < 0.01 (Mann-Whitney test, 3 independent cultures). (G) Representative images of DIV8, DIV10 WT mouse cortical neurons electroporated with L315 control construct (Control) or L315 Nrxn triple knockdown construct (Nrxns TKD) immunostained for pan-Nrxn (grayscale) and EGFP (grayscale). Blue [file pbio.3000466.s007.tif]

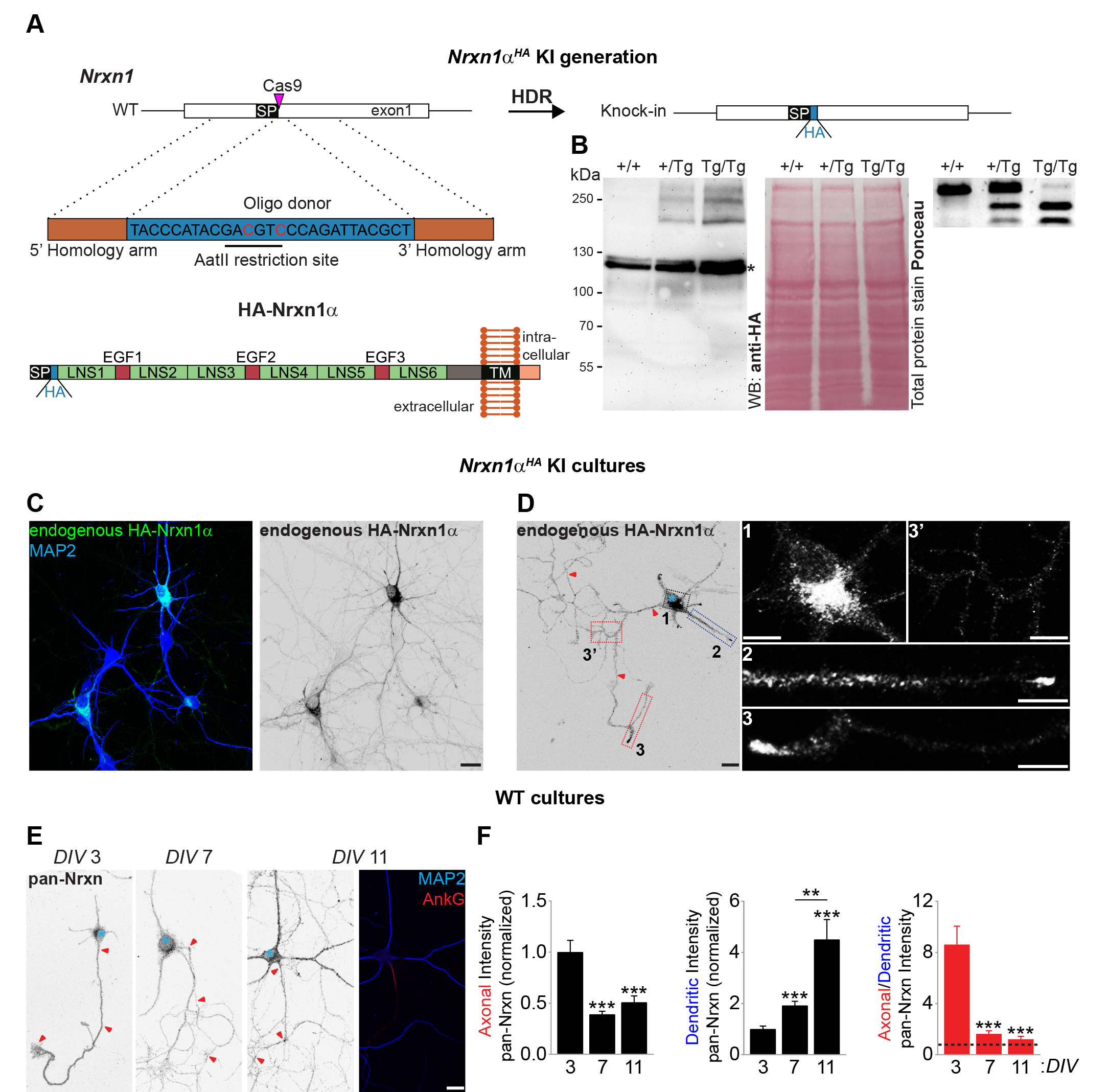

Supplement: S2 Fig — (A) Orange boxes represent the left and right homology arms. Blue box represents the ssDNA donor oligonucleotide containing the HA tag. CRISPR/Cas9-mediated HDR allowed for precise HA-tagging of the Nrxn1 locus right after the SP. An AatII restriction site was introduced in the DNA sequence coding for the HA tag, by taking advantage of the redundancy of the genetic code, in order to facilitate the distinction between homozygous and heterozygous Nrxn1αHA mice. Schematic representation of Nrxn1α protein domain organization is shown to illustrate the HA-tagging of HA-Nrxn1α right after the SP in the extracellular domain. (B) Left panels: detection of HA-Nrxn1α by western blot in cortical extracts prepared from adult Nrxn1αHA KI mice (3 months old). Total protein staining by using Ponceau method was used as loading control. Asterisk indicates an aspecific band. Right panel: identification of WT (+/+), heterozygous (+/Tg), and homozygous (Tg/Tg) Nrxn1αHA mice by generation of a PCR fragment of 499 bp followed by AatII-mediated restriction endonuclease reaction generating 2 DNA fragments of 312 bp and 187 bp. See S9 Fig for raw uncropped blots. (C and D) Representative images of permeabilized DIV7 Nrxn1αHA mouse cortical neurons cultured together with WT mouse cortical neurons and immunostained for HA-Nrxn1α (green and grayscale) and MAP2 (blue). Red arrowheads indicate the axon, and the blue asterisk marks the cell body. High-zoom images of the subcellular distribution of endogenous HA-Nrxn1α: cell body (1), dendrites (2) and axon (3 and 3ʹ). (E) Permeabilized DIV3, DIV7, and DIV11 WT mouse cortical neurons labeled with a pan-Nrxn antibody (grayscale). MAP2 (blue) and Ankyrin-G (AnkG; red) identify somatodendritic and axonal compartments, respectively. (F) Quantification of panel E: pan-Nrxn fluorescence intensity in axon and dendrites normalized to DIV3 neurons and ratio of axonal–dendritic pan-Nrxn intensity. DIV3 (n = 30 neurons); DIV7 (n = 30); DIV11 (n = 28). **P < [file pbio.3000466.s008.tif]

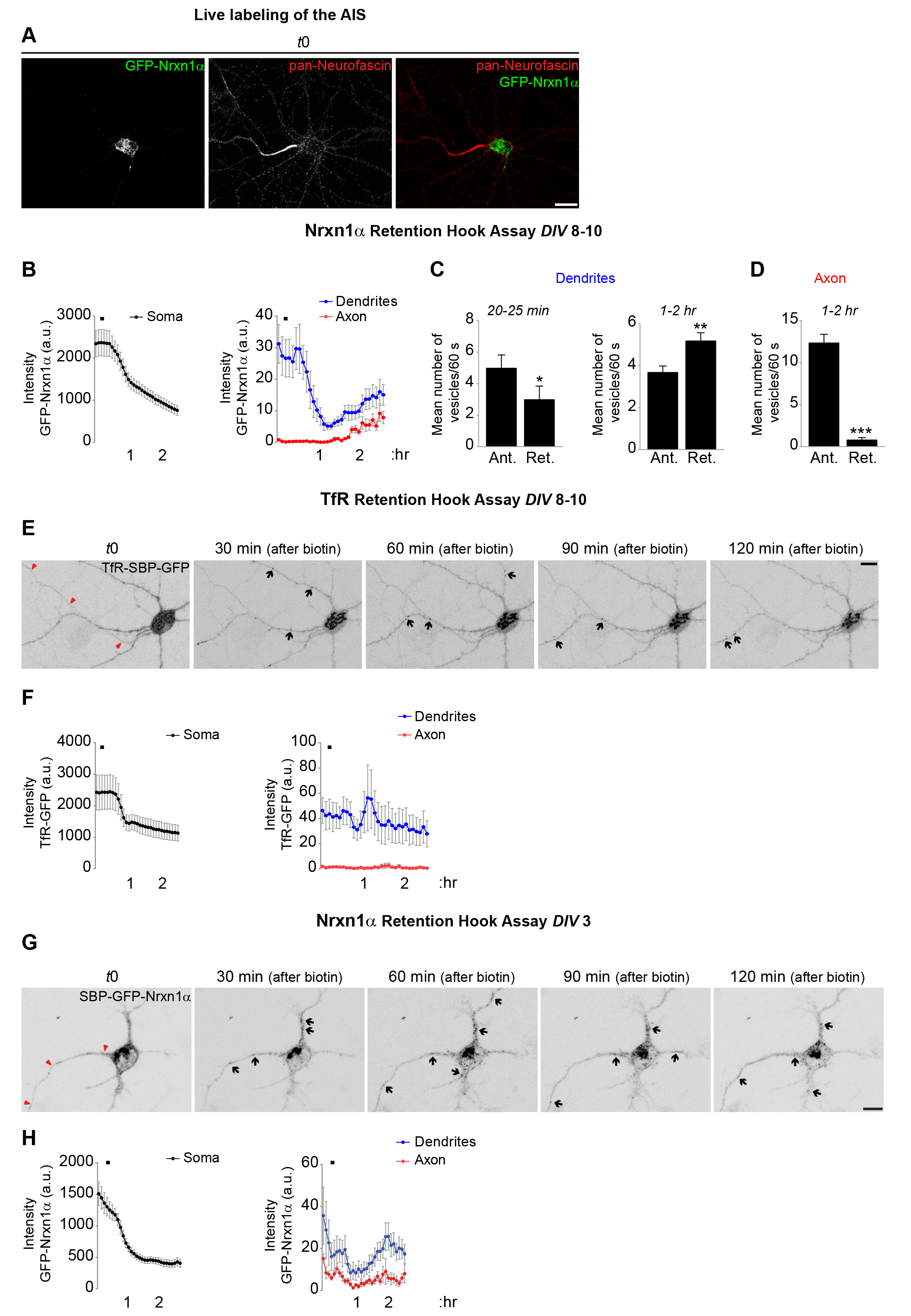

Supplement: S3 Fig — (A) Illustration of the strategy used during live-cell imaging experiments to label the axon. Representative image of a live DIV8 WT rat cortical neuron co-expressing SBP-EGFP-Nrxn1α (grayscale and green) and streptavidin-KDEL immunostained for pan-Neurofascin (axon initial segment marker, grayscale and red). (B) Quantification of SBP-EGFP-Nrxn1α (n = 23 neurons) fluorescence intensity in soma, dendrites and axon in 3 independent experiments. (C and D) Mean number of SBP-EGFP-Nrxn1α vesicles moving in anterograde and retrograde direction in (C) dendrites and (D) axons. In dendrites, analysis was performed at 2 different time intervals: 20 to 25 min (n = 8 neurons), and 1 to 2 h (n = 16) after adding biotin; in axons, analysis was performed 1 to 2 h after biotin (n = 16). *P < 0.05; **P < 0.01; ***P < 0.001 (Mann-Whitney test). (E) Live-cell imaging in DIV8 to DIV10 WT rat cortical neurons co-expressing TfR-SBP-EGFP and ER hook. After 24 to 31 h of expression, neurons were imaged every 5 min for 2.5 h. Biotin was added 10 min after the beginning of the imaging session. Shown are representative images of TfR-SBP-EGFP fluorescence in dendrites and axon before (t0) and 30, 60, 90, and 120 min after adding biotin. Red arrowheads indicate axon, and black arrows indicate TfR-SBP-EGFP-positive puncta. See also S4 Movie. (F) Quantification of TfR-SBP-EGFP (n = 13 neurons) fluorescence intensity in soma, dendrites and axon in 3 independent experiments. (G) Live-cell imaging in DIV3 WT rat cortical neurons co-expressing SBP-EGFP-Nrxn1α and streptavidin-KDEL. After 25 to 29 h of expression, neurons were imaged every 5 min for 2.5 h. Biotin was added 10 min after the beginning of the imaging session. Shown are representative images of EGFP-Nrxn1α endogenous fluorescence in dendrites and axons before (t0), 30, 60, 90, and 120 min after adding biotin. Red arrowheads indicate the axon and black arrows indicate EGFP-Nrxn1α-positive puncta. See also S6 Movie. (H) Quantification of EG [file pbio.3000466.s009.tif]

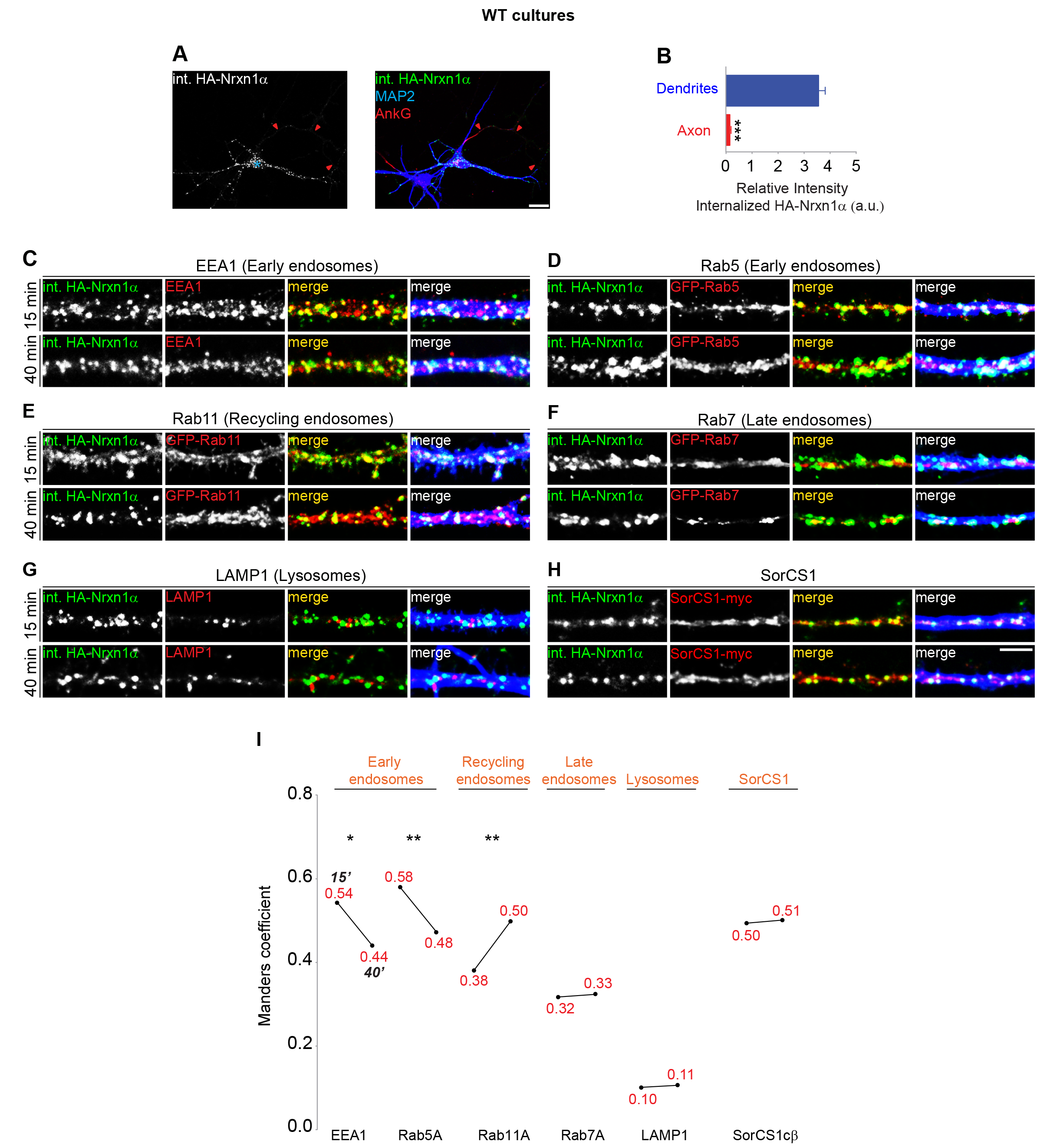

Supplement: S4 Fig — (A–I) Antibody pulse-chase experiments in DIV8 to DIV11 WT mouse cortical neurons expressing extracellular HA-tagged Nrxn1α or co-expressing HA-Nrxn1α and endosomal markers for 48 h. (A) Representative images of mouse cortical neurons pulse-chased for 15 min and immunostained for internalized HA-Nrxn1α (grayscale and green), MAP2 (blue), and Ankyrin-G (red). Red arrowheads indicate the axon, and the blue asterisk marks indicate the cell body. (B) Quantification of internalized Nrxn1α fluorescence intensity in dendrites and axons relative to total levels (n = 15 neurons). ***P < 0.001 (Mann-Whitney test, 3 independent experiments). Graph shows mean ± SEM. (C–H) High-zoom images of dendritic segments from mouse cortical neurons pulse-chased for 15 min or 40 min and immunostained for internalized HA-Nrxn1α (grayscale and green), MAP2 (blue); EEA1 (EE marker, in grayscale and red) (C), EGFP-Rab5 (EE marker, in grayscale and red) (D), EGFP-Rab11 (RE marker, in grayscale and red) (E), EGFP-Rab7 (late endosomal marker, in grayscale and red) (F), LAMP1 (lysosomal marker, in grayscale and red) (G) or SorCS1-myc (in grayscale and red) (H). (I) Quantification of the colocalization of internalized Nrxn1α with different endosomal or lysosomal markers expressed as Mander’s coefficient (n = 15 neurons for each group). Graph shows mean. *P < 0.05; **P < 0.01 (Mann-Whitney test, 3 independent experiments). Underlying numerical values can be found in S1 Data. Scale bars, 20 μm (panel A); 5 μm (panel H). DIV, days in vitro; EE, early endosome; EEA1, early endosome antigen 1; HA, hemagglutinin; LAMP1, lysosomal-associated membrane protein 1; MAP2, microtubule-associated protein 2; Nrxn, neurexin; Rab, Rab GTPase; RE, recycling endosome; SorCS1, Sortilin-related CNS expressed 1; WT, wild type. (TIF) [file pbio.3000466.s010.tif]

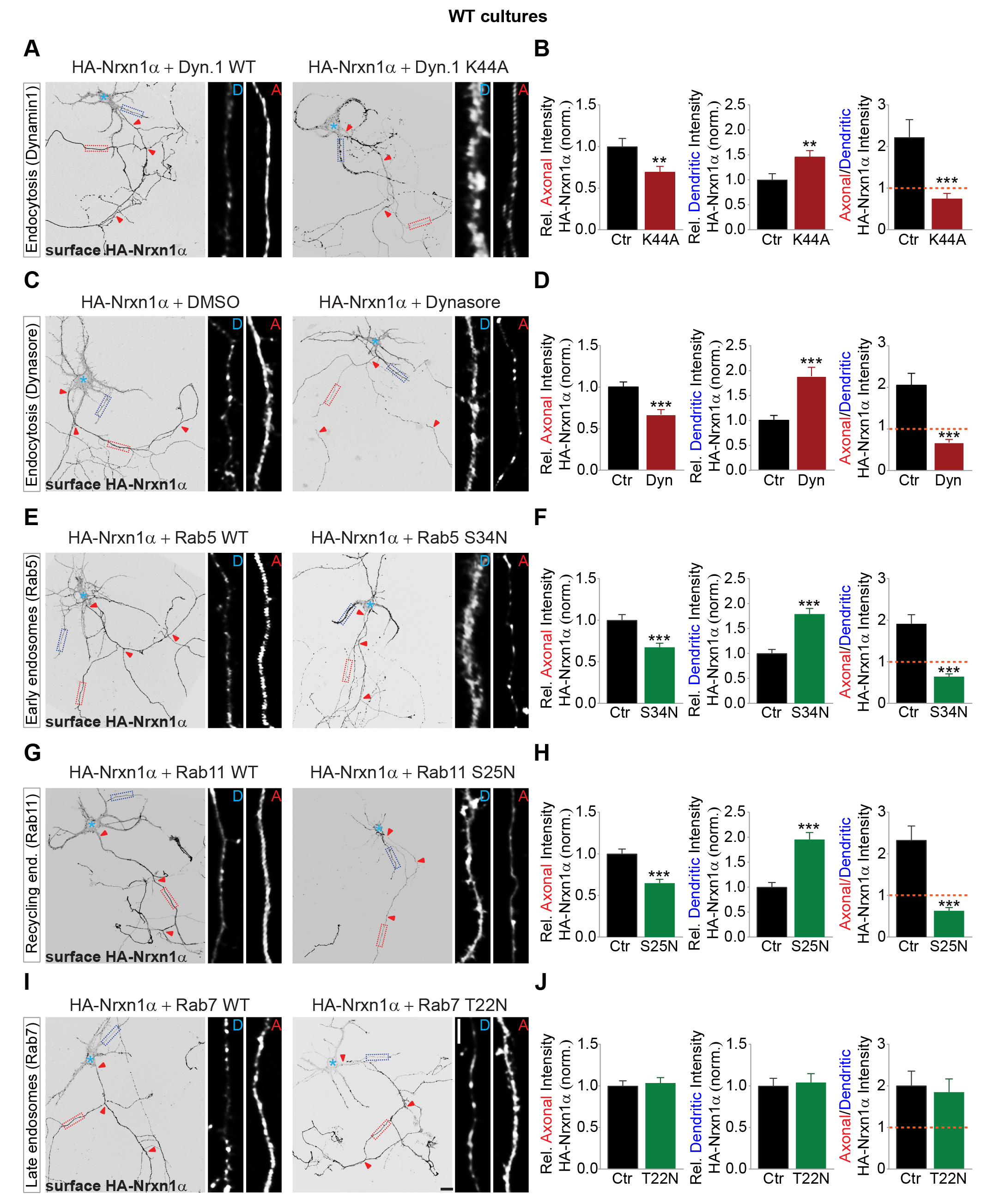

Supplement: S5 Fig — (A) Representative images of DIV8 to DIV10 WT mouse cortical neurons co-expressing HA-Nrxn1α and EGFP-tagged WT Dynamin1 (Ctr) or a dominant negative of EGFP-Dynamin1 (K44A, to block Dynamin-dependent endocytosis) for 48 h. Neurons were immunostained for surface HA-Nrxn1α in grayscale. Red arrowheads indicate the axon, and the blue asterisk marks the cell body. High-zoom images of dendritic (D, dotted blue box) and axonal (A, dotted red box) Nrxn1α are shown next to the whole-cell images. (B) Quantification of panel A: surface HA-Nrxn1α fluorescence intensity in axon and dendrites relative to total surface levels and normalized to cells expressing Dyn.1 WT and ratio of axonal–dendritic surface HA intensity. Ctr (n = 30 neurons); K44A (n = 30). (C) Representative images of DIV8 to DIV9 WT mouse cortical neurons expressing extracellular HA-tagged Nrxn1α and treated either with DMSO (vehicle, Ctr) or with Dynasore (Dyn) 30 h after transfection. Neurons were immunostained 18 h after treatment for surface HA-Nrxn1α (grayscale). (D) Quantification of panel C; Ctr (n = 29 neurons); Dyn (n = 25). (E) Representative images of DIV8 to DIV10 WT cortical neurons co-expressing HA-Nrxn1α and EGFP-tagged WT Rab5 (Ctr) or a dominant negative of EGFP-Rab5 (S34N, to prevent the formation of EEs) for 48 h. Neurons were immunostained for surface HA-Nrxn1α (grayscale). (F) Quantification of panel E; Ctr (n = 29 neurons); S34N (n = 27). (G) Representative images of DIV8 to DIV10 WT cortical neurons co-expressing HA-Nrxn1α and EGFP-tagged WT Rab11 (Ctr) or a dominant negative of EGFP-Rab11 (S25N, to prevent the formation of REs) for 48 h. Neurons were immunostained for surface HA-Nrxn1α (grayscale). (H) Quantification of panel G; Ctr (n = 30 neurons); S25N (n = 26). (I) Representative images of DIV8 to DIV10 WT mouse cortical neurons co-expressing extracellular HA-tagged Nrxn1α and EGFP-tagged WT Rab7 (Ctr) or a dominant negative of EGFP-Rab7 (T22N, to prevent the formation of late endoso [file pbio.3000466.s011.tif]

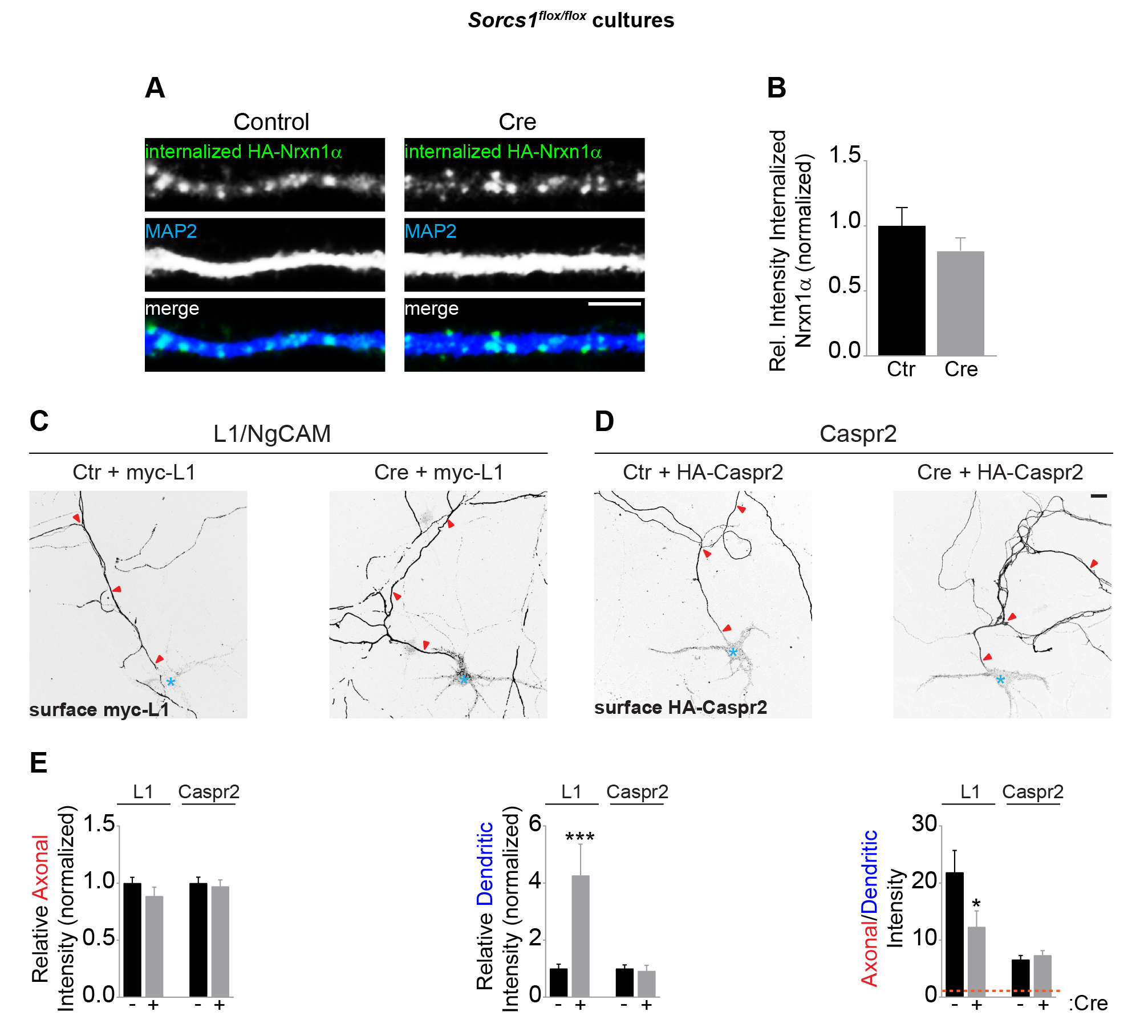

Supplement: S6 Fig — (A) High-zoom representative images of dendritic internalized Nrxn1α from DIV8, DIV10 Sorcs1flox/flox cortical neurons electroporated with EGFP (Control) or Cre-EGFP (Cre) and transfected with extracellular HA-tagged Nrxn1α. After 48 h, neurons were live-labeled with anti-HA antibody and pulse-chased for 20 min, followed by immunostaining for internalized HA-Nrxn1α (grayscale and green) and MAP2 (grayscale and blue). (B) Quantification of panel A: internalized Nrxn1α fluorescence intensity relative to total levels and normalized to cells expressing EGFP (3 independent experiments). Control (n = 30 neurons); Cre (n = 28). (C and D) Representative images of DIV8 to DIV10 Sorcs1flox/flox mouse cortical neurons electroporated with EGFP (Ctr) or Cre-EGFP, and transfected with myc-L1 (C) or HA-Caspr2 (D) for 48 h, followed by immunostaining for surface myc-L1 or HA-Caspr2 in grayscale. (E) Quantification of panels C and D: surface myc-L1 and HA-Caspr2 fluorescence intensity in axon and dendrites relative to total surface levels and normalized to cells expressing EGFP and ratio of axonal–dendritic surface L1 and Caspr2 intensity. Ctr_L1 (n = 29 neurons); Cre_L1 (n = 26); Ctr_Caspr2 (n = 28); Cre_Caspr2 (n = 25). *P < 0.05; **P < 0.01; ***P < 0.001 (Mann-Whitney test, 4 independent cultures). Underlying numerical values can be found in S1 Data. Graphs show mean ± SEM. Scale bars, 5 μm (panel A), 20 μm (panel D). Caspr2, contactin-associated protein-like 2; Cre, Cre recombinase; Ctr, control; DIV, days in vitro; EGFP, enhanced green fluorescent protein; HA, hemagglutinin; MAP2, microtubule-associated protein 2; Nrxn, neurexin; SorCS1, Sortilin-related CNS expressed 1. (TIF) [file pbio.3000466.s012.tif]

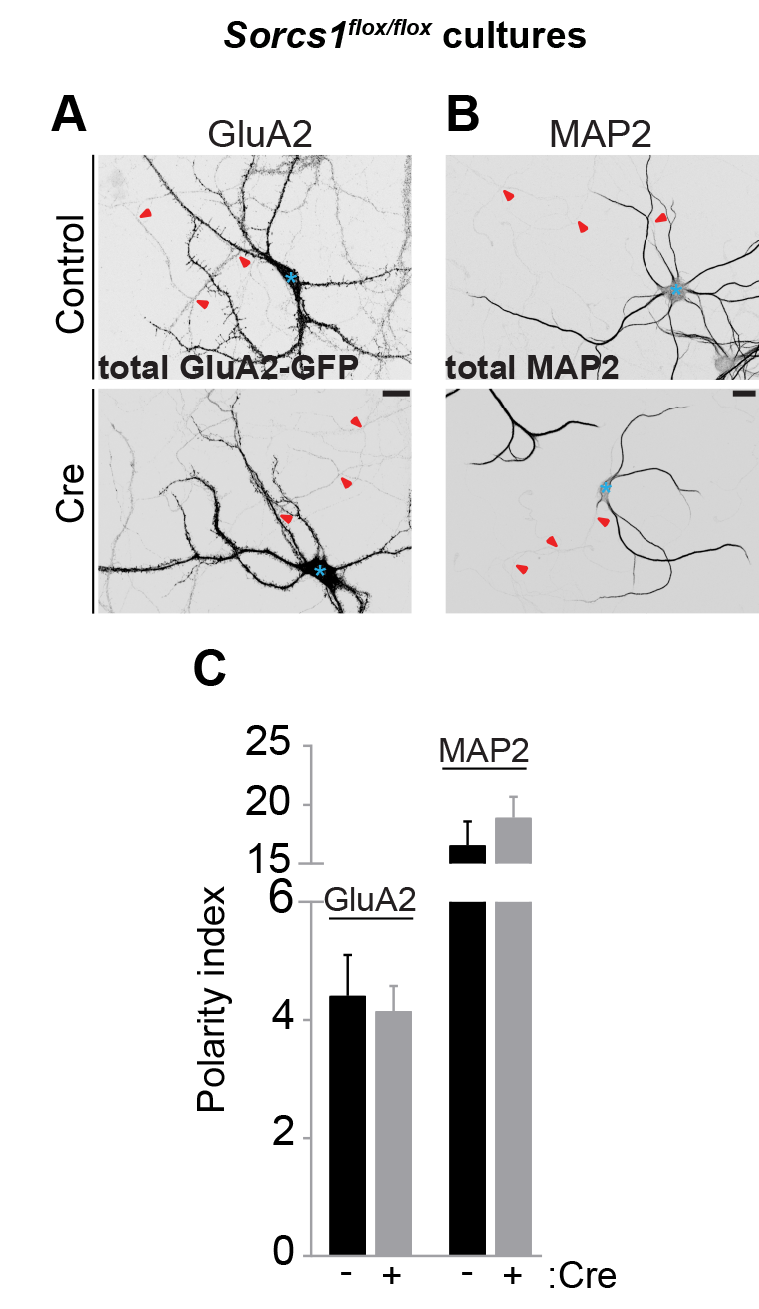

Supplement: S7 Fig — (A) Representative images of DIV14 Sorcs1flox/flox cortical neurons co-expressing EGFP-GluA2 and an empty vector (Ctr) or EGFP-GluA2 and Cre-myc (Cre) for 7 d. Neurons were immunostained for EGFP-GluA2 (grayscale). Red arrowheads indicate the axon, and the blue asterisk marks the cell body. (B) Representative images of DIV10 mouse Sorcs1flox/flox cortical neurons electroporated with EGFP (Ctr) or Cre-EGFP (Cre) immunostained for MAP2 (grayscale). (C) Quantification of panels A and B: dendritic versus axonal distribution (D:A–polarity index) of GluA2 or MAP2 in WT and Sorcs1 KO cells (at least 1 independent experiment). GluA2_Ctr (n = 9 neurons); GluA2_Cre (n = 9); MAP2_Ctr (n = 18); MAP2_Cre (n = 20). Underlying numerical values can be found in S1 Data. Graph shows mean ± SEM. Scale bars, 20 μm. Cre, Cre recombinase; Ctr, control; DIV, days in vitro; EGFP, enhanced green fluorescent protein; GluA2, glutamate ionotropic receptor AMPA type subunit 2; KO, knock out; MAP2, microtubule-associated protein 2; SorCS1, Sortilin-related CNS expressed 1; WT, wild type. (TIF) [file pbio.3000466.s013.tif]

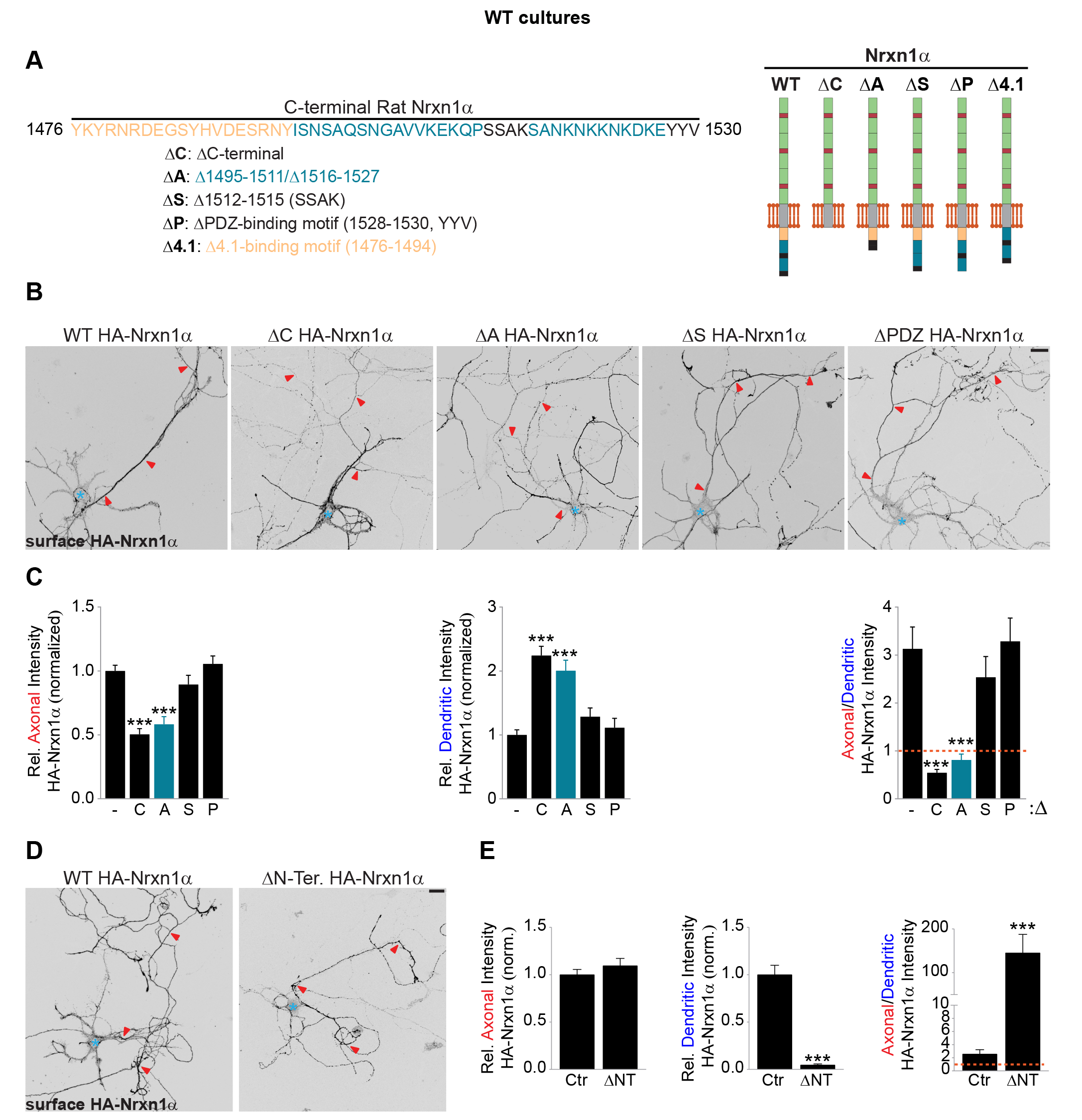

Supplement: S8 Fig — (A) Schematic representation of WT and C-terminal mutants of rat Nrxn1α. Different deletion mutants of Nrxn1α C terminal were designed: deletion mutant of the entire C terminal (ΔC), deletion mutant spanning the regions 1495–1511 and 1516–1527 (ΔA), deletion mutant lacking a putative phosphorylation site in serine 1513 (ΔS), deletion mutant lacking the PDZ-binding motif (ΔP), and deletion mutant lacking the 4.1- binding motif (Δ4.1). (B) Representative images of DIV8 to DIV10 WT mouse cortical neurons expressing extracellular HA-tagged WT-Nrxn1α and C-terminal deletions mutants of Nrxn1α for 48 h and immunostained for surface HA-Nrxn1α (grayscale). (C) Quantification of panel B: surface HA-Nrxn1α fluorescence intensity in axon and dendrites relative to total surface levels and normalized to cells expressing WT-Nrxn1α and ratio of axonal–dendritic surface HA intensity. WT (n = 40 neurons); ΔC (n = 30); ΔA (n = 30); ΔS (n = 28), ΔP (n = 28). ***P < 0.001 (Kruskal-Wallis test followed by Dunn’s multiple comparisons test, at least 3 independent experiments). (D) Representative images of DIV8 to DIV10 WT mouse cortical neurons expressing extracellular HA-tagged WT-Nrxn1α and N-terminal deletion mutant of Nrxn1α for 48 h and immunostained for surface HA-Nrxn1α (grayscale). (E) Quantification of panel D: surface HA-Nrxn1α fluorescence intensity in axon and dendrites relative to total surface levels and normalized to cells expressing WT-Nrxn1α and ratio of axonal–dendritic surface HA intensity. WT (n = 30 neurons); ΔNT (n = 29). ***P < 0.001 (Mann-Whitney test, 3 independent experiments). Underlying numerical values can be found in S1 Data. Graph shows mean ± SEM. Scale bars, 20 μm. DIV, days in vitro; HA, hemagglutinin; Nrxn, neurexin; PDZ, PSD-95/discs large/zona occludens-1 motif; WT, wild type. (TIF) [file pbio.3000466.s014.tif]

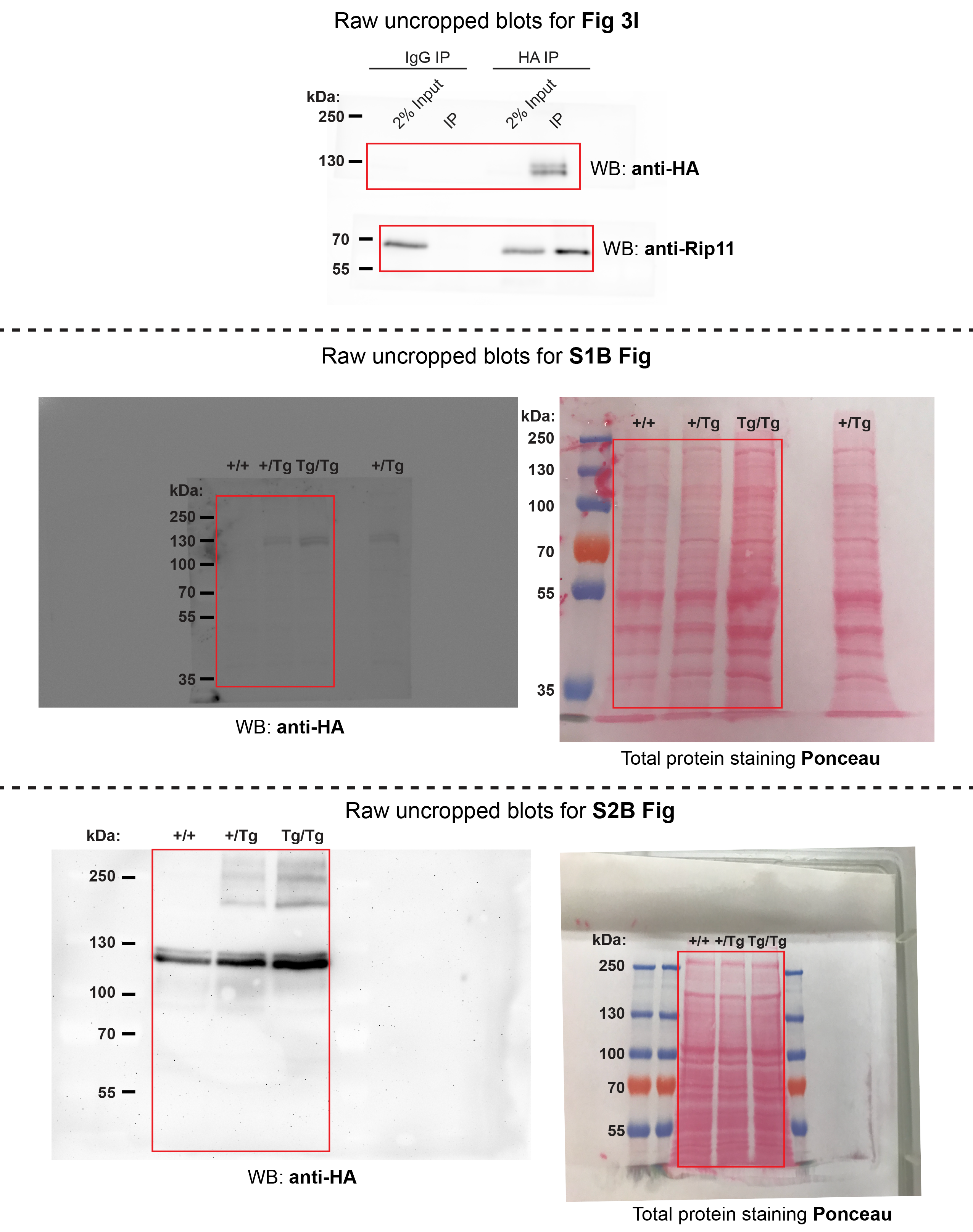

Supplement: S9 Fig — Raw uncropped blots for Fig 3I, S1B Fig, and S2B Fig. (TIF) [file pbio.3000466.s015.tif]
