# Supplementary material for: SorCS1-mediated sorting in dendrites maintains neurexin axonal surface polarization required for synaptic function
Source: PLoS Biol. 2019 Oct 28;17(10):e3000466. doi: 10.1371/journal.pbio.3000466 (PMC6837583; doi:10.1371/journal.pbio.3000466)
Supplement: Attachment — Submitted filename: Response to Reviewers PBIOLOGY-D-19-01893R1.docx [file pbio.3000466.s019.docx]

We thank the reviewers for their time and effort, and appreciate their positive and constructive feedback on our manuscript. We have adjusted the text to meet the reviewers’ concerns, which improved the manuscript considerable. Below is detailed point-by-point reply to address individual reviewer comments.

**Response to Reviewer #1**

Point 1. *Can the authors describe the localization of Neurexin relative to other endosomal markers (as shown in Fig. 3) for additional time points than those shown? I am not proposing to show all the time points for all the RABs.  But it seems that they should mention whether the time point shown are the only ones a particular RAB showed SorCS1 dependent behaviour.*

We agree with the reviewer that this an important point to address. We have now analyzed the colocalization of internalized Nrxn1α with a series of markers and SorCS1. To follow the fate of internalized Nrxn1α, neurons were incubated with an anti-HA antibody, chased for 15 min or 40 min, and immunostained for internalized HA-Nrxn1α and a panel of markers for different endosomal compartments, lysosomes and SorCS1 in dendrites. We observed a decrease in colocalization of internalized Nrxn1α with early endosomes (EEs; EEA1- and Rab5-positive), and an increase in colocalization with recycling endosomes (REs; Rab11-positive) in cells chased for 40 min compared to 15 min. These results suggest a Nrxn1α protein flux from the dendritic plasma membrane to EEs and subsequently to REs. On the other hand, at these two different time points, internalized Nrxn1α colocalizes abundantly with SorCS1, which suggest that internalized Nrxn1α localizes to SorCS1-positive puncta when it transits from the dendritic plasma membrane to REs. This data has been added to S4 Fig (S4C-I Fig) and the text has been adapted to include these results:

Internalized Nrxn1α was largely restricted to the dendritic compartment (S4A and S4B Fig) and shifted from early endosomes (EEs; EEA1- and Rab5-positive) to recycling endosomes (REs; Rab11-positive) over time (S4C–I Fig).

Point 2*. There is a typo on Figure 2 (RE lumen instead of ER lumen).*

We thank the reviewer for noticing this. This typo has been corrected.

Point 3. *The text and Figure S3 refer to neurofascin as the axonal marker, but the corresponding main figure (Fig 2D) shows AnkG.*

In Fig 2D we indeed showed AnkG as an axonal marker, but in our live-cell imaging experiments all neurons were previously live-labeled with an anti-pan-Neurofascin antibody to distinguish the axon from the dendrites. The anti-AnkG antibody is not suitable for the live-labeling procedure, but this protocol works nicely with the anti-pan-Neurofascin antibody. In Fig 2D we wanted to illustrate the accumulation of Nrxn1α in the endoplasmic reticulum without biotin application in fixed neurons. On the other hand, in S3 Fig we wanted to illustrate the strategy of live-labeling with the anti-pan-Neurofascin antibody used to label the axonal compartment. We apologize for the confusion and have now moved this panel to the upper part of S3 Fig (S3A Fig), which will hopefully highlight the differences between these two panels.

Point 4. *The first two sentences of the ‘Nrxn1α is transcytosed’ section are confusing based on how it is presently written. The previous section showing the RUSH data seems to answer the question asked in these two sentences.*

We thank the reviewer for pointing this out. In order to address this comment and avoid any confusion these two sentences have been changed:

Our observations suggest a dendrite-to-axon transcytotic pathway for axonal surface polarization of Nrxn1α. The transcytotic model predicts that Nrxn1α is first trafficked to the somatodendritic plasma membrane, where it is internalized into endosomes and trafficked to the axonal compartment via endosome-derived carriers [36]. To determine whether Nrxn1α is internalized from the dendritic plasma membrane, we followed the fate of Nrxn1α throughout the endosomal pathway.

**Response to Reviewer #2**

Point 1. *In respect to the previous work, the transcytosis of aNrxn1 in cortical neurons seem to be the major process in synapse formation and function and does only little affect postsynaptic AMPAR-density and function. The formation and function of synapses depend on the here investigated trafficking of aNrxn1 but seem not being affected by postsynaptic changes in AMPAR trafficking, as proposed by Savas et al. 2015. Indeed, the probed parameters indicate a strong effect of SOR-CS1 KO on presynaptic functions, but are their remaining increased numbers of postsynaptic silent synapses, as discussed? May the autaptic cultures bias the effect on AMPARs by homeostatic upregulation of receptor density? Are their data for the surface population of AMPARs in these autaptic cultures, which could support the idea of postsynaptic AMPAR-silent synapses.*

As correctly pointed out by the reviewer we have previously found that excitatory synapse density is unaffected in *Sorcs1* KO neurons, but the fraction of silent synapses lacking functional AMPARs is increased in cultured neurons (Savas et al., Neuron, 2015). In this current manuscript, we extended our original analysis and probed for pre-synaptic defects in autaptic *Sorcs1* KO cells. Consistent with a pre-synaptic defect, depletion of SorCS1 produced a more pronounced rundown of normalized evoked responses (synaptic depression) compared to control conditions during train stimulations. Moreover, we also found that the amplitude of evoked EPSCs and the readily releasable pool (RRP) size were decreased in *Sorcs1* KO neurons, which can be explained either by the presynaptic defect or by the increase in the number of silent excitatory synapses. Although back-extrapolation analysis of cumulative synchronous charge during 10 Hz-train stimulation confirmed a presynaptic defect independent of the silent synapses phenotype in *Sorcs1* KO neurons (Fig 5K), we cannot rule out an effect on silent synapses density in autaptic *Sorcs1* KO cells. We did not generate new data to address this important point, but we have added a sentence to the Discussion to highlight it:

Our results show that loss of SorCS1 in autaptic cortical neurons decreases mEPSC frequency, eEPSC amplitude, and sucrose-evoked responses, in line with the reported decreased fraction of active synapses in *Sorcs1* KO neurons [20]. Whether this decrease in the fraction of active synapses is also happening in autaptic cultured neurons requires further experimental testing. Furthermore, we found an increase in synaptic depression of normalized eEPCSs during stimulus trains, similar to alpha-Nrxn KO neurons [7].

Point 2. *The mentioned dendritic sorting motives in page 13, second paragraph are probably wrong spelled (“YxxO and [D/E]xxx[L/I] dendritic sorting motifs [48] but….”)*

We thank the reviewer for pointing this out. We corrected the ‘tyrosine-based’ consensus motif in the text for YXXØ (where Ø is an amino acid with a bulky hydrophobic side chain, i.e. leucine, isoleucine, methionine, valine, or phenylalanine), and spelled the ‘dileucine-based’ in its most used form: (D/E)*XXX*L(L/I). The text has been updated accordingly.

**Response to Reviewer #3**

Point 1.  *Fig. 3E, why the number of Nrxn punta is so few compared to Fig. 3A and 3C in the Cre expressing conditions? In Fig. 3G, is the number of Nrxn punta decreased in Cre expressing conditions in axons?*

In Fig 3E the number of Nrxn puncta overlapping with Rab11 is decreased in *Sorcs1* KO cells because upon deletion of SorCS1 transition of internalized Nrxn1α from EEs to REs is compromised in dendrites (Fig 3E). This leads to a severe decrease in the number of double positive puncta for internalized Nrxn1α and Rab11 in axons (Fig 3G), in support of the idea that Nrxn1α trafficking from dendrites to axons in Rab11-positive endosomes is regulated by SorCS1. We hope this clarifies the reviewer’s comment.

Point 2. *Is the expression of SorCS1 or Rip11 developmentally regulated?*

We agree with the reviewer that this an interesting point to address, especially because of the differences in Nrxn1α trafficking described in our manuscript for mature and immature neurons. Unfortunately, to our knowledge, for Rip11 this has never been addressed before. On the other hand, *Sorcs1* expression has been analysed with *in situ* hybridization in the developing and adult mouse nervous system (Oetjen et al., Journal of Comparative Neurology, 2014). In case of the cerebral cortex, SorCS1 is detectable from embryonic day 18, and is present during the development and in the adult. However, from the published data it is hard to deduce whether the expression of SorCS1 is developmentally regulated or not, because the available data lacks quantitation. Therefore we can unfortunately not speculated whether the expression of SorCS1 or Rip11 are developmentally regulated, and if this correlates with the differences in Nrxn1α trafficking during development. We have added a sentence to the Discussion to reflect this point:

As neurons mature, Nrxn1α follows an indirect trafficking pathway after TGN exit via SorCS1-mediated sorting in dendritic endosomes and transcytosis to the axonal surface. It will be interesting to determine whether expression of *Sorcs1* and *Rip11* is developmentally regulated in these neurons. The biological function of this developmental regulation and circuitous trafficking route is not clear.
